# Gleanings from Our Exchanges

**Published:** 1874-02-01

**Authors:** 


ON CLEFT PALATE.—T. P. Pick, Esq., records (St. George's Hospital Reports) eleven cases of this defect, with an account of the operation in each. In connection with the subject of hæmorrhage, Mr. Pick says a free amount of bleeding, during the operation, is rather a favorable symptom than otherwise; that in those cases where the bleeding is free, union will be found much more perfect than where the parts are anæmic and bleed more slightly. The hæmorrhage, he adds, is rarely so excessive as to produce any serious effect, either on the union of the wound or the health of the patient. Mr. P. does not favor the early operation, but believes it advisable to delay cutting as long as possible; *i. e.*, "as long as there is no fear of the child's acquiring defective articulation." Silk sutures were used for the soft palate, and silver for the hard, Mr. P. thinking the former more manageable than any other, more easily introduced, more readily secured, and much less likely to slip. He uses a perfectly pure silk, plaited, instead of the ordinary twist. He allows the sutures to remain for eight days, and, except in a single instance, when one of them produced a little irritation, has found no inconvenience from them. The after-treatment consists in giving as much *fluid* nourishment as the patient will take, with a fair allowance of wine, Mr. P. believing that, to obtain good union, it is of the first importance to keep up the patient's strength.—*Amer. Practitioner.*

---

DEVELOPMENT OF CANCER OF THE SKIN.—(*Amer. Jour. of Syph. and Derm.*, from *Virchow's Archiv*). According to Carmalt, who has examined three carcinomatous tumors of the skin, the epitheleum of the hair follicles is the point of departure of the cancerous growth, which throws some light upon the cause of cancer of the skin. Fuhrer states that frequent and rough shaving is apt to produce cancer of the skin on the face. Out of 50 or 60 cases of cancer of the lip and cheek, occurring, recently, in the Breslau Pathological Institute, only two were in women, and not one case among men with unshaved beards. Carmalt supports the view of Waldeyer and others, regarding the historical origin of cancer, that every cancerous growth originates in the epithelial elements of the part, which opposes Virchow's views, that the cancer-cells are the equivalents of connective tissue corpuscles.—*Boston Med. and Surg. Jour.*

